# A clinical study evaluating low dose ferrous fumarate vs. standard iron supplements in iron-deficient non-anemic to mild anemic adults

**DOI:** 10.1038/s41598-024-65878-5

**Published:** 2024-07-08

**Authors:** G. S. Jyothi, Rohit Shelatkar, H. R. Kalavathy, V. G. Vaidya, Manjit Sisode, Gayatri Ganu

**Affiliations:** 1 Ramaiah Medical College, Bengaluru, Karnataka 560054 India; 2 Vitabiotics Ltd, 1 Apsley Way, London, NW2 7HF UK; 3 Kala Hospital and Clinical Laboratory, 1105, KN Extension, 5Th Cross, Triveni Road, Yeswanthpur, Bengaluru, Karnataka 560022 India; 4 Lokmanya Medical Research Centre and Hospital, Fourth Floor OPD 401314 B Telco Road, Chinchwad, Pune, Maharashtra India; 5 Janseva Hospital, 2Nd Floor, Datta Mandir Chowk, Deopur, Dhule India; 6 Mprex Healthcare Pvt. Ltd., Office Number 501, 514 Crossroads, Bhumkar Square, Wakad, Pune, India

**Keywords:** Iron deficiency anemia, Hemoglobin, Ferritin, Ferrous fumarate, Non-anemic, Health care, Medical research, Signs and symptoms

## Abstract

Our study aims to validate safety and efficacy of Feroglobin capsule compared with different iron supplementations in adult subjects diagnosed with non-anemic to mild anemic iron deficiency and fatigue. Enrolled 302 participants diagnosed with non-anemic to mild anemic iron deficiency and fatigue. Group A (*n* = 147) received Feroglobin, Group B (*n* = 146) received standard of care [Haem Up Gems capsules (Ferrous fumarate) or Fericip tablets (Ferrous ascorbate)]. 293 subjects completed the study with follow-up visits on days 30, 60, and 90. Feroglobin treatment significantly increased hemoglobin levels from mean 12.43 g/dl to 13.24 g/dl in 90 days. Ferritin levels improved significantly by 442.87% compared to the standard care’s 256.67%. Fatigue scale scores reduced by 47.51%, and all presenting health complaints resolved completely. Gastrointestinal symptoms observed were similar in both the groups. Both groups exhibited moderate treatment adherence. Quality of life improved in pain and general health domains, exhibiting a good tolerability. Adverse events were unrelated to the investigational products. Feroglobin serves as an efficacious therapeutic alternative for improving hemoglobin, ferritin, and reducing fatigue with low doses compared to standard of care. However, longer-term effects of low-dose require further investigations in different target groups.

## Introduction

Iron deficiency is a prevailing cause of nutritional deficiency^1^. The Global Burden of Disease Study 2021 reported that IDA is the leading cause of anemia, accounting for 66.2% of all anemia cases. According to a 2021 report, 825 million women and 444 million men worldwide are affected by iron deficiency anemia^2^.

It is often associated with nonspecific complaints such as fatigue, a decreased ability to work, pallor, shortness of breath, weakness, lightheadedness, palpitations worsening congestive heart failure, and dyspnea on exertion. The causes of IDA vary according to age, gender, and socioeconomic status. Iron deficiency usually results from insufficient iron intake, decreased absorption (e.g. celiac disease), increased systemic requirements (e.g. pregnancy, adolescent age), and depletion of iron stores (e.g. blood loss, depleted ferritin)^3,4^. In the human body, 60% of iron is in hemoglobin (heme iron), 30% is stored as ferritin and hemosiderin, and the remaining 10% is a part of cytochrome enzymes or myoglobin. Iron is a micronutrient essential for the binding and transport of oxygen, the storage of iron, and the regulation of cellular respiration. Iron is an essential constituent of mitochondrial enzymes and myoglobin; thus, iron deficiency may result in fatigue and weakness before hemoglobin levels decrease^1^.

The initial phase of anemia occurs when iron stores in the body are depleted, resulting in hypo-ferritinemia but hemoglobin (Hb) is still within the normal range; this is referred to as non-anemic iron deficiency (NAID)^5^. Undetected and untreated iron deficiency anemia (IDA) can have a considerable impact on individual health. Chronic iron deficiency affects general well-being; and leads to fatigue and reduced working capacity. Indeed, accumulating evidence also suggests a significant correlation between iron deficiency and DNA replication and the cell cycle (oral lesions, hair loss, nail abnormalities), altered immune response (increase susceptibility to infections), and restless leg syndrome, and inhibition of cytochrome P450 production may result in altered drug metabolism, maternal sepsis, hemorrhage, and maternal and child mortality^6,7^.

Successful iron replacement therapy (IRT) should be able to replenish body iron stores, improve QoL and physiological functions, and restore normal Hb levels^5^. The treatment of IDA includes oral and intravenous (IV) IRT and blood transfusion.

The patient's age and sex, the underlying cause, severity, and symptoms of IDA, and the available or acceptable time frame for correction need to be considered when deciding treatment^7^. Oral iron therapy remains the mainstay of treatment for IDA, and is convenient, inexpensive, and effective for treating stable patients. The most commonly prescribed iron salt supplements are sulfate, gluconate, fumarate, ascorbate, or carbonyl iron.

Long-term treatment with oral iron is limited by intolerance associated with metallic taste, and gastrointestinal symptoms such as nausea, vomiting, constipation, and abdominal discomfort. This leads to frequent dose adjustments, changes in prescription, no adherence, or treatment discontinuation^7,8^. Although intravenous iron is more reliably and quickly distributed than oral iron, its primary disadvantages are higher cost, practical inconvenience, and limited access. However, it is also associated with adverse effects such as nausea, anaphylaxis, and extravasation^4^.

Oral iron therapy is a well-established and effective treatment for anemia due to its efficacy, safety, and cost-effectiveness. However, there is a lack of clinical trials comparing different iron preparations, dosages, and durations of therapy. Despite being standard care, oral iron therapy still has unmet needs concerning the type of preparation, dosage, and control of side effects^9^. It is crucial to determine the most appropriate form and dose of iron as well as the duration of treatment to successfully replenish iron stores, returning Hb to a normal level with minimal side effects in a more assured and appropriate way. To address these gaps, we propose a study comparing slow-release, low-dose ferrous fumarate with standard ferrous fumarate.

Feroglobin capsules a special slow-release delivery system designed to enhance the gradual and even release of ferrous fumarate along with essential vitamins and minerals into the bloodstream. The gelatin capsule disintegrates in the stomach within one minute. The polymer coating of hydroxypropyl methylcellulose (HPMC) and ethylcellulose (EC) aids in the slow release of minerals. The technology used in the manufacturing of Feroglobin capsules offers This coating facilitates controlled release in the stomach over a period of two hours, preventing dose dumping and reducing gastric irritation thus other side effects. As a result, the bioavailability of iron is higher, and patient compliance is improved. These features make Feroglobin capsules a potentially better-tolerated and more effective option compared to standard iron supplements^10^.

The present research is an attempt at clinical validation of the safety and effectiveness of the Feroglobin capsule with lower dose of elemental iron compared with different iron supplementation models in reducing fatigue and improving iron stores and Hb in subjects with non-anemic to mild anemic iron deficiency and suffering from fatigue.

## Materials and methods

### Study design

This was a multicenter, longitudinal, prospective, cohort, clinical study to evaluate the efficacy and safety of different iron supplementations in adults who had non-anemic to mild anemic iron deficiency and who were experiencing fatigue. The treatment group received a Feroglobin capsule, while the standard of care received either Haem Up Gems capsules or Fericip tablets. The duration of the treatment period was 90 days.

### Inclusion criteria

The study recruited adult individuals aged 18–65 years, inclusive, who exhibited fatigue within the range of 18 to 54 on the Fatigue Severity Scale (FSS) at screening. Eligible participants had serum ferritin levels less than 50 mcg/L, which is indicative of iron deficiency with hemoglobin levels equal to or greater than 11 g/dL for non-pregnant women and men, as per the World Health Organization's criteria for mild anemia to non-anemic patients. Participants, both with and without comorbidities, were included, provided they had been on stable prescriptions for the preceding three months. Eligible individuals were required to provide written informed consent and complete follow-up visits throughout the study period.

### Exclusion criteria

Individuals who met any of the following criteria were deemed ineligible for participation in the study: having previously been diagnosed with any concurrent disease, medical, or surgical condition that could contribute to fatigue or iron deficiency; a medical history indicating current hematological disorders other than iron deficiency anemia, such as aplastic anemia, megaloblastic anemia, sideroblastic anemia, pernicious anemia, thalassemia, or sickle cell anemia; a medical history of malabsorption syndrome, hypochlorhydria, achlorhydria, gastrectomy, or gastrojejunostomy; eligibility for intravenous iron replacement therapy; documented significant internal or external bleeding; recent treatment with vitamin and mineral supplements within the past three months; undergoing dialysis or having an estimated glomerular filtration rate less than 30 mL/minute/1.73 m^2^; a current or past history of malignancy within the preceding three years; being pregnant, planning pregnancy, breastfeeding, within four weeks postpartum, or having a positive pregnancy test; known or suspected hypersensitivity to iron or any investigational product components; and lacking competence to complete study questionnaires as determined by the investigator's discretion.

### Sample size

A sample size of total 274 completed cases needed to assess the study objective (reduction of mean FSS score by at least 5% by test group compared to placebo) at 95% power and 5% level of significance.

The allocation of subjects in Test: Placebo is 1:1 hence we require 137 subjects in test group and placebo respectively to attain the 95% power and 5% level of significance.

### Methodology

**Figure 1:**
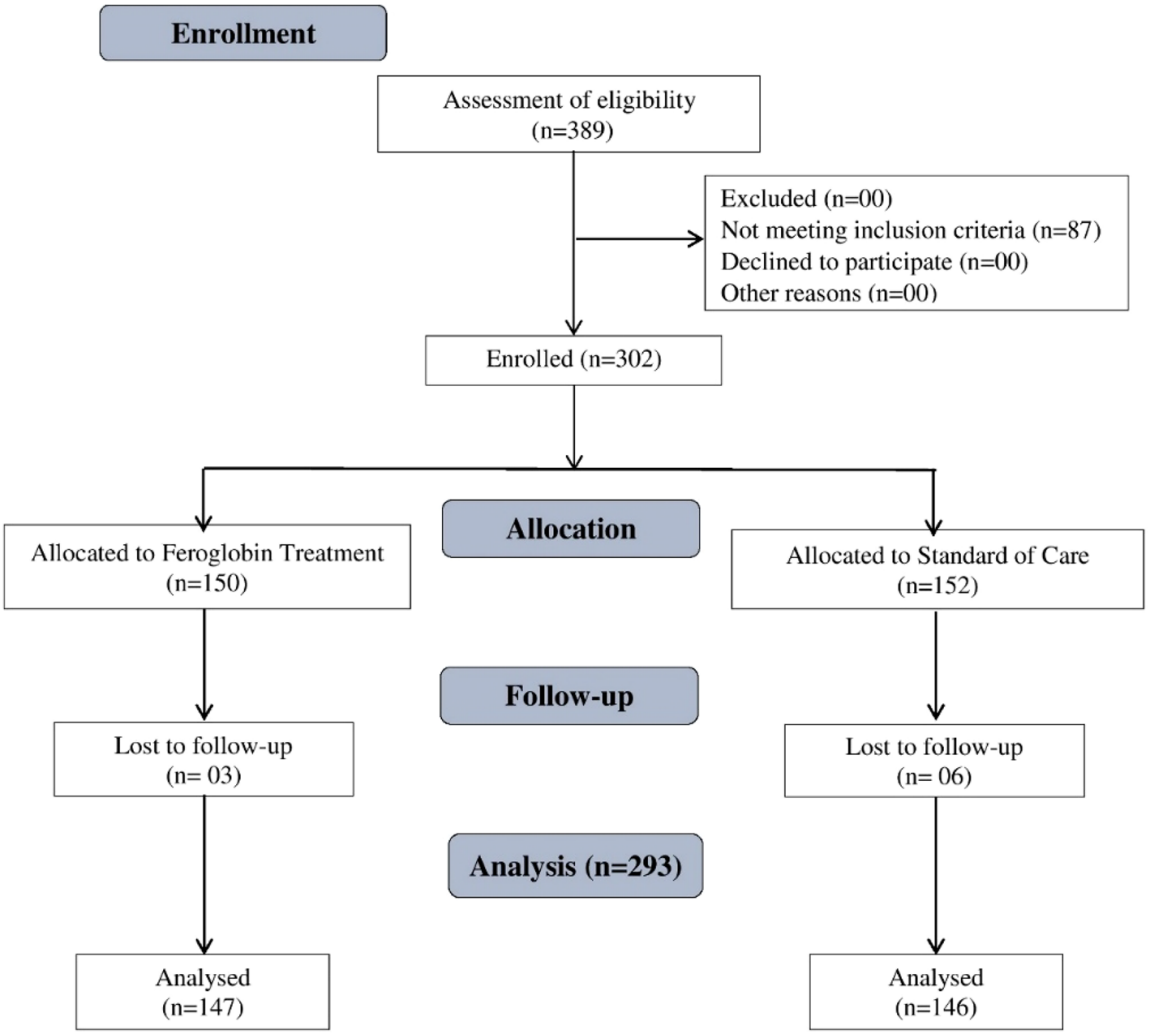
CONSORT diagram for the study.

In this study, 300 subjects were assessed throughout a treatment duration of 90 days. The subjects were equally randomized using computer generated randomization sheet to either the Feroglobin or standard of care treatment group (150 in each arm). The safety and efficacy of different oral iron supplementations, in doses prescribed by each investigator in line with the standard practices of each center, were compared with those of the Feroglobin treatment capsule regimen in the study subjects. Efficacy assessments were conducted at each visit (Days 30, 60, and 90). The two groups were monitored both clinically, and for adverse reactions linked with it. Assessment of fatigue severity score, hemoglobin, ferritin level, and gastrointestinal adverse effects were assessed on screening, days 30, 60, and 90. Assessment of quality of life was performed on screening and day 90. Treatment adherence was assessed every month. The safety and tolerability of the investigational treatment in terms of Adverse Events (AEs), and Serious Adverse Events (SAEs) were assessed from the baseline visit to day 90. The consort diagram is depicted in Fig. 1.

### Statistical analysis

Statistical analysis was performed using SPSS version 10.0. The primary and secondary endpoints were analyzed using a Student t-test, Wilcoxon signed rank test, and Mann–Whitney U Test. The *p*-values ≤ 0.05 were considered to indicate statistical significance.

### Ethical approval

Ethical approval for the study was obtained from the Institutional Ethics Committee (IEC) of Lokmanya Medical Research Centre, Chinchwad, and the Royal Pune Independent Ethics Committee. The clinical trial was registered with the Clinical Trial Registry-India (CTRI) under the registration number CTRI/2023/01/048,783 on 05/01/2023. URL: https://ctri.nic.in/Clinicaltrials/pmaindet2.php?EncHid=NzgwNTk=&Enc=&userName=CTRI/2023/01/048783. The study was conducted as per the approved protocol, declaration of Helsinki and Good Clinical Practices guidelines. Clinical trial data were collected between April 2023 and November 2023. The compositions of the investigational products are depicted in Table 1.

**Table 1:** Investigational product and standard of care product composition.

Investigational product	Standard of care	
Feroglobin group capsule	Haem up gems capsule	Fericip tablet
Elemental Iron (Ferrous fumarate) (24 mg)	Ferrous fumarate (200 mg)	Ferrous ascorbate (100 mg)
Folic Acid (500 mcg)	Folic Acid (1.5 mg)	Folic Acid (1.5 mg)
Elemental Copper (2 mg)	Cupric Sulfate (2.5 mg)	–
Vitamin B6 (5 mg)	Manganese sulfate (2.5 mg)	–
Elemental Zinc (12 mg)	–	–
Vitamin B12 (10 mcg)	–	–

## Results

### Demographic characteristics

In this study, a total of 300 male and female subjects were initially enrolled, with 293 completing the study. Among the participants, 46 males and 101 females were in the Feroglobin treatment, and 33 males and 113 females were in the standard of care. The average age among Feroglobin treatment was 36.10 ± 11.79 years, while among the standard of care, it was 34.47 ± 11.41 years. The details are presented in Table 2. There were five subjects with a medical history of comorbidities, three of whom had diabetes mellitus and two of whom had hypertension. All the subjects who had comorbidities were on stable prescription.

**Table 2:** Demographic details.

Parameter	Feroglobin (*n* = 147)	Std. of Care (*n* = 146)	Total	*P* value
Male	46	33	79	0.093751
Female	101	113	214	
Total	147	146	293	
Average ± SD Age	36.10 ± 11.79	34.47 ± 11.41	–	

The data was analyzed by chi-square test (significant at *p* < 0.05).

### Assessment of changes in hemoglobin levels and ferritin levels

In this study, hemoglobin levels gradually and significantly improved among Feroglobin treatment subjects by 3.13%, 6.13%, and 5.69% after day 30, day 60, and day 90, respectively. Whereas, in standard of care hemoglobin levels improved by 1.14%, 2.19%, and 3.04% after day 30, day 60, and day 90, respectively. The significant improvement was relatively greater in Feroglobin treatment compared to standard of care. However, the comparison between the groups was not significant but within the group was significant as depicted in Table 3.

**Table 3:** Comparison of hemoglobin levels and ferritin levels between the groups.

Duration (Days)	Feroglobin (*n* = 147)	Std. of Care (*n* = 146)	*P* value
Mean Hemoglobin (gm/dl) (Average ± SD)			
Screening	12.43 ± 1.16 (10.8–16.5)	12.49 ± 1.15 (10.9–16.4)	0.424
Day 30	12.82 ± 1.37* (9.8–17.4)	12.64 ± 1.20 (10–16.6)	0.121
Day 60	13.19 ± 1.44* (10.2–17.2)	12.77 ± 1.25* (10–16.6)	0.002
Day 90	13.13 ± 1.46* (11–17.1)	12.87 ± 1.35* (8.9–16.9)	0.535
Mean Ferritin (ng/ml) (Average ± SD)			
Screening	26.27 ± 12.71 (1.25–49.76)	24.66 ± 12.78 (2.4–49)	0.271
Day 30	53.41 ± 21.21* (12.6–110)	41.07 ± 20.4* (9.3–148.1)	0.00001
Day 60	98.21 ± 34.19* (18.2–198)	66.77 ± 25.6* (11.9–196.7)	0.00001
Day 90	142.61 ± 63.73* (23.6–293)	87.94 ± 33.3* (13.3–196)	0.00001

The data was analyzed by the Mann–Whitney U Test between groups. Within-Feroglobin analysis was carried out by Wilcoxon Signed-Ranks Test. (significant at *p* < 0.05; within group significance denoted as *).

As illustrated in Table 3, the ferritin level exhibited a gradual and statistically significant increase among subjects receiving Feroglobin treatment. This increased ferritin levels to 103.30%, 273.86%, and 442.87% on days 30, 60, and 90, respectively. According to the standard of care, the ferritin level increased by of 66.55%, 170.79%, and 256.67% on day 30, day 60, and day 90, respectively. The significant improvement was relatively greater in the Feroglobin treatment group compared to standard of care group. The differences between the groups and within the groups were statistically significant.

### Assessment of the fatigue severity score

**Figure 2:**
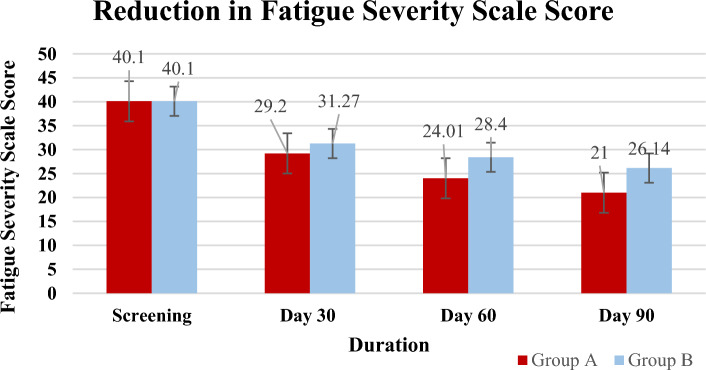
Reduction in Fatigue Severity Scale Score.

As shown in Fig. 2, at screening, the FSS score was 40.1 among Feroglobin treatment, which was comparable to the 40.10 among standard of care, and the difference was not statistically significant. The fatigue severity score of the Feroglobin treated group significantly decreased to 29.20, 29.20, and 21, respectively, while in the standard of care, the FSS score decreased to 31.27, 28.40, and 26.14 at days 30, 60, and 90, respectively. The percent changes after 30 days, 60 days, and 90 days of treatment significantly decreased by 27%, 39.99%, and 47.51%, respectively, among Feroglobin treatment, compared to respective percent reduction of 22.03%, 29.17%, and 34.81% in the standard of care group.

### Assessment of treatment adherence

Treatment adherence was assessed using the Morisky Medication Adherence Scale-8 (MMAS-8), with scores ranging from 0 to 8 points, where higher scores indicate better adherence. Adherence levels were categorized as low (< 6 points), moderate (6–7 points), or high (8 points). Treatment adherence was found as 6.20, 6.76, and 6.80 points in the Feroglobin treatment, respectively. Similarly, in the standard of care, treatment adherence was 6.34, 6.57, and 6.68 after 30 days, 60 days, and 90 days, respectively (Table 4). Both groups demonstrated moderate treatment adherence. Additionally, all subjects from both groups exhibited good tolerability.

**Table 4:** Comparison of treatment adherence between the groups.

Duration (Days)	Treatment adherence (MMAS-8) (Average ± SD)	
	Feroglobin (*n* = 147)	Std. of Care (*n* = 146)
Day 30	6.20 ± 1.41	6.34 ± 1.26
Day 60	6.76 ± 1.01	6.57 ± 1.06
Day 90	6.80 ± 1.07	6.68 ± 0.99

### Assessment of gastrointestinal symptoms

In this study, we evaluated various symptoms experienced by the patients over a period of 90 days. These symptoms included constipation, nausea, vomiting, metallic taste, and abdominal pain/discomfort. To assess the severity of these symptoms, we used a 4-point ordinal scale as follows: 0—None: No symptoms reported; 1—Mild: Symptoms were present but did not interfere with daily activities; 2—Moderate: Symptoms were moderate and interfered with daily activities; 3—Severe: Symptoms were severe and significantly interfered with daily activities. The severity of these symptoms at four time points: baseline, day 30, day 60, and day 90.

There were some gastrointestinal symptoms observed in both groups. There were no statistically significant changes observed in gastrointestinal symptoms, such as constipation, nausea, vomiting, metallic taste, and abdominal pain/discomfort, in both groups throughout the 90 days between groups and magnitude wise, similar symptoms in the Feroglobin and standard of care group than standard of care (Table 5).

**Table 5:** Assessment of gastrointestinal symptoms.

Parameter	Visits	Screening		Day 30		Day 60		Day 90	
	Score	Feroglobin	Std	Feroglobin	Std	Feroglobin	Std	Feroglobin	Std
Constipation	0	70	69	28	26	33	17	36	39
	1	49	45	65	60	68	76	80	80
	2	28	32	54	56	46	53	31	27
	3	0	0	0	4	0	0	0	0
	*P* values	0.951	0.784	0.014	0.663				
Nausea	0	108	124	132	132	139	135	143	143
	1	30	17	14	14	8	11	4	3
	2	9	5	1	0	0	0	0	0
	3	0	0	0	0	0	0	0	0
	P values	0.016	0.860	0.467	0.709				
Vomiting	0	133	133	137	134	140	138	143	143
	1	13	13	10	12	7	8	4	3
	2	1	0	0	0	0	0	0	0
	3	0	0	0	0	0	0	0	0
	*P* values	0.855	0.646	0.781	0.709				
Bad Test	0	135	127	135	135	141	138	141	145
	1	7	17	12	10	6	8	6	1
	2	5	2	0	1	0	0	0	0
	3	0	0	0	0	0	0	0	0
	*P* values	0.177	0.841	0.574	0.057				
Abdominal Pain/Discomfort	0	76	74	43	39	55	40	62	53
	1	45	44	54	51	44	53	50	68
	2	26	28	49	52	48	53	35	25
	3	0	0	1	4	0	0	0	0
	*P* values	0.862	0.628	0.0670	0.303				
Bloating	0	54	53	46	42	42	44	44	52
	1	55	36	29	28	42	35	44	29
	2	31	44	58	51	37	46	48	55
	3	7	13	14	25	26	21	11	9
	*P* values	0.939	0.637	0.769	0.281				

All the data was analyzed by chi-square test (significant at *p* < 0.05 denoted as *).

### Assessment of presenting health complaints

The following prevalent health complaints, tiredness, weakness, low energy, and headache, were demonstrated by subjects in both groups at screening. Notably, all of the presenting health complaints were completely resolved (100%) (subject reported outcome) within 90 days of the study duration in both groups.

### Assessment of quality of life (QoL)

The assessment of the QoL score was conducted using the SF-36 questionnaire, with questions divided into eight domains: physical functioning, limitations due to physical health, limitations due to emotional problems, energy/fatigue, emotional well-being, social functioning, pain domain, and general health domain.

After 90 days, Feroglobin treatment significantly improved the pain and general health QoL domains compared to the standard of care (*p* < 0.05). Additionally, comparable improvements were observed in the remaining six QoL domains (Table 6).

**Table 6:** Assessment of Changes in Quality of Life Score using SF-36 questionnaire.

Assessment of Changes in Quality of Life Score			
Duration (Days)	Mean Total Score (Average ± SD)		*P* value
	Feroglobin (N = 147)	Std. of care (N = 146)	
Physical functioning			
Screening	637.76 ± 268.02	637.67 ± 273.90	0.936
Day 90	897.65 ± 154.70*	886.99 ± 169.28*	0.555
Limitations due to physical health			
Screening	246.26 ± 117.20	236.30 ± 118.54	0.497
Day 90	334.01 ± 78.07*	322.60 ± 87.71*	0.358
Limitations due to emotional problems			
Screening	182.99 ± 82.24	180.82 ± 74.57	0.741
Day 90	253.06 ± 54.02*	246.58 ± 63.42*	0.596
Energy/Fatigue			
Screening	186.80 ± 55.47	184.93 ± 57.85	0.928
Day 90	214.97 ± 33.26*	213.84 ± 44.73*	0.795
Emotional well-being			
Screening	325.99 ± 75.76	315.48 ± 79.96	0.184
Day 90	319.73 ± 54.90*	321.10 ± 58.71*	0.549
Social functioning			
Screening	134.01 ± 57.48	132.88 ± 59.14	0.889
Day 90	132.38 ± 28.78*	127.91 ± 31.83*	0.242
Pain			
Screening	141.94 ± 49.97	141.71 ± 53.87	0.857
Day 90	150.82 ± 33.84*	140.31 ± 35.33*	0.005
General health			
Screening	308.91 ± 75.84	306.03 ± 80.59	0.575
Day 90	361.05 ± 51.03*	339.04 ± 63.31*	0.0008

Data was analyzed by the Mann–Whitney U Test between groups. Within-group analysis was carried out by Wilcoxon Signed-Ranks Test (significant at *p* < 0.05).

### Assessment of adverse events

Ten subjects in Feroglobin treatment group experienced adverse events compared to nine subjects in the standard of care group. The adverse events reported included headaches, wound cuts, coughing, and minor burns. Additionally, all adverse events in both groups were resolved by providing the required medications during the study period. None of the adverse events were related to the investigational products. These findings suggest that Feroglobin treatment and standard of care exhibited comparable safety profiles, with no significant differences in the incidence or types of adverse events observed between the two groups (Table 7).

**Table 7:** Assessment of Adverse Events.

Adverse events	Feroglobin		Std. of care	
	No. of AE	%	No. of AE	%
Headache	4	2.72	3	2.05
Wound cut	2	1.36	2	1.37
Cough	3	2.04	4	2.74
Minor burn	1	0.68	0	0.00
Total no. of events	10	6.80	9	6.16
Total no. of patients	10	6.80	9	6.16

## Discussion

In this study, the effectiveness of the Feroglobin treatment capsule was compared with different iron supplementations in adult subjects who were diagnosed with non-anemic to mild anemic iron deficiency and who were suffering from fatigue. After a 90-day treatment period, statistically significant improvements were observed in the mean hemoglobin and ferritin levels in both the groups, but it was more among the Feroglobin treatment subjects compared to standard of care. Moreover, the study found that the treatment with Feroglobin was significantly more effective in reducing the severity of fatigue compared to standard of care. Treatment adherence was assessed based on the Morisky Medication Adherence Scale-8 (MMAS-8), and both groups demonstrated moderate treatment adherence. Furthermore, subjects exhibited good tolerance to the investigational products. No statistically significant changes were observed in gastrointestinal symptoms though magnitude-wise there were fewer gastrointestinal effects in the Feroglobin treated group. The presenting health complaints were resolved completely till day 90. Quality of life was assessed based on the SF-36 questionnaire which is divided into 8 domains. A statistically significant improvement was observed in the pain and general health QoL domains compared to the standard of care. Additionally, statistically non-significant but, comparable improvements were observed in the remaining six QoL domains. All the adverse events observed among both study groups during the study were not related to the investigational products and were resolved by providing the rescue medications.

Jongkraijakra et al. showed that a lower ferrous fumarate dose along with less frequent administration is more effective, with less incidence of adverse effects^11^. Rimon et al. reported that ninety elderly hospitalized patients with iron-deficiency anemia received either elemental iron 15 mg or 50 mg as liquid ferrous gluconate or 150 mg of ferrous calcium citrate tablets daily for 60 days. Results showed that two low-dose schedules were equally effective as high traditional doses in the treatment^12^.

A crossover study conducted by Stoffel et al. demonstrated that women with IDA, who received either 100 or 200 mg iron as ferrous sulfate on consecutive days and alternate days. The results indicate that alternate-day dosing results in higher fractional iron absorption and a trend toward a lower incidence of gastrointestinal side effects compared to consecutive-day dosing^13^. Moretti et al. reported that, after oral iron administration of more than 60 mg (BD or OD), serum hepcidin increases and affects fractional iron absorption. Lower dosages (40–80 mg) of elemental iron and the avoidance of twice-daily dosing maximize fractional iron absorption^14^. Similarly, the present study demonstrated that a dose of Feroglobin, which contains a lower amount of elemental iron (24 mg) and folic acid, which is one-tenth of the recommended comparative products, still efficiently increased hemoglobin and ferritin levels in subjects without producing substantial adverse effects. Additionally, GI symptoms observed were similar to the standard of care group along with good tolerability. However, the longer-term effects of low-dose Feroglobin require further investigations in different target groups.

Our study results confirm and add to the findings of a randomized controlled study conducted by Verdon and colleagues, who observed improvements in fatigue among non-anemic women with unexplained fatigue following iron supplementation^15^. Similarly, Vaucher et al. reveal that oral iron supplementation demonstrated improvements in fatigue, hemoglobin, ferritin, hematocrit, mean corpuscular volume, and soluble transferrin within six weeks of initiating treatment^16^.

Furthermore, our study provided validated evidence-based data about the safety and effectiveness of investigational products. It will help determine the most appropriate, formulation, dose, and duration of treatment for non-anemic to mild anemic iron deficiency and associated fatigue.

This research helps address important medical needs in safely and effectively treating non-anemic to mild anemic iron deficiency and reducing related fatigue. The research outcomes of this study will be instrumental for further research in the same field and will serve as a guiding light for clinical practice.

There is a need to perform translational work focused on some targeted aims and come up with a treatment model for the same to supply adequate iron to replenish body iron stores, improve quality of life and physiologic function, and reduce symptoms of IDA, especially fatigue. This could be achieved through the present research. These research outcomes will be utilized to develop a strategy for treating non-anemic to mild anemic iron deficiency and fatigue in adult patients in a more appropriate and assured way.

## Conclusion

This study demonstrated that Feroglobin serves as an efficacious therapeutic alternative for improving hemoglobin levels, ferritin levels, and fatigue with low doses compared to the recommended standard of care. However, the longer-term effects of low-dose Feroglobin require further investigations in different target groups. Furthermore, Feroglobin demonstrates similar incidences of gastrointestinal symptoms and the absence of substantial adverse effects related to investigational products. Importantly, Feroglobin effectively enhances overall patient quality of life. The evidence-based data presented in this study reaffirm the safety and efficacy of Feroglobin, offering a promising therapy for the management of non-anemic to mild anemic iron deficiency.

## Data Availability

The datasets generated and/or analysed during the current study are not publicly available due Intellectual property constraints but are available from the corresponding author on reasonable request.

## References

[1] Świątczak M (2022). Chronic fatigue syndrome in patients with deteriorated iron metabolism. Diagnostics.

[2] GBD 2021 Anaemia Collaborators (2023). Prevalence, years lived with disability, and trends in anaemia burden by severity and cause, 1990–2021: findings from the Global Burden of Disease Study 2021. The Lancet Haematol..

[3] Anaemia in women and children. WHO 2019. https://www.who.int/data/gho/data/themes/topics/anaemia_in_women_and_children

[4] Warner, M. J. & M. T. Kamran. Iron Deficiency Anemia. StatPearls, StatPearls Publishing, 7 (2023). 28846348

[5] Snook J (2021). British Society of Gastroenterology guidelines for the management of iron deficiency anaemia in adults. Gut.

[6] Paul BT (2017). Mitochondria and Iron: current questions. Expert Rev. Hematol..

[7] Cappellini MD (2020). Iron deficiency anaemia revisited. J. Internal Med..

[8] Anaemia—World Health Organization (WHO). https://www.who.int/health-topics/anaemia

[9] Russo G, Guardabasso V, Romano F (2020). Monitoring oral iron therapy in children with iron deficiency anemia: an observational, prospective, multicenter study of AIEOP patients (Associazione Italiana Emato-Oncologia Pediatrica). Annals Hematol..

[10] Santiago P (2012). Ferrous versus ferric oral iron formulations for the treatment of iron deficiency: a clinical overview. Sci. World J..

[11] Jongkraijakra S (2023). A randomized controlled trial of thrice-weekly versus thrice-daily oral ferrous fumarate treatment in adult patients with iron-deficiency anemia. Annals Hematol..

[12] Rimon E (2005). Are we giving too much iron? Low-dose iron therapy is effective in octogenarians. Am. J. Med..

[13] Stoffel NU (2020). Iron absorption from supplements is greater with alternate day than with consecutive day dosing in iron-deficient anemic women. Haematologica.

[14] Moretti D (2015). Oral iron supplements increase hepcidin and decrease iron absorption from daily or twice-daily doses in iron-depleted young women. Blood.

[15] Verdon F (2003). Iron supplementation for unexplained fatigue in non-anaemic women: double blind randomised placebo controlled trial. BMJ (Clinical research ed.).

[16] Vaucher P (2012). Effect of iron supplementation on fatigue in nonanemic menstruating women with low ferritin: a randomized controlled trial. Can. Med. Assoc. J..

